# NMDA Receptor in Vasopressin 1b Neurons Is Not Required for Short-Term Social Memory, Object Memory or Aggression

**DOI:** 10.3389/fnbeh.2019.00218

**Published:** 2019-11-08

**Authors:** Sarah K. Williams Avram, Heon-Jin Lee, Jarrett Fastman, Adi Cymerblit-Sabba, Adam Smith, Matthew Vincent, June Song, Michael C. Granovetter, Su-Hyun Lee, Nicholas I. Cilz, Michelle Stackmann, Rahul Chaturvedi, W. Scott Young

**Affiliations:** ^1^Section on Neural Gene Expression, National Institute of Mental Health, National Institutes of Health, Bethesda, MD, United States; ^2^Systems Neuroscience Imaging Resource, Intramural Research Program, National Institute of Mental Health, National Institutes of Health, Bethesda, MD, United States; ^3^Department of Microbiology and Immunology, School of Dentistry, Kyungpook National University, Daegu, South Korea; ^4^Neuroscience Program, Department of Pharmacology & Toxicology, University of Kansas, Lawrence, KS, United States

**Keywords:** vasopressin, Avpr1b, NMDA, aggression, social memory, CA2, hippocampus, social interaction

## Abstract

The arginine vasopressin 1b receptor (Avpr1b) plays an important role in social behaviors including aggression, social learning and memory. Genetic removal of Avpr1b from mouse models results in deficits in aggression and short-term social recognition in adults. Avpr1b gene expression is highly enriched in the pyramidal neurons of the hippocampal cornu ammonis 2 (CA2) region. Activity of the hippocampal CA2 has been shown to be required for normal short-term social recognition and aggressive behaviors. Vasopressin acts to enhance synaptic responses of CA2 neurons through a NMDA-receptor dependent mechanism. Genetic removal of the obligatory subunit of the NMDA receptor (Grin1) within distinct hippocampal regions impairs non-social learning and memory. However, the question of a direct role for NMDA receptor activity in Avpr1b neurons to modulate social behavior remains unclear. To answer this question, we first created a novel transgenic mouse line with Cre recombinase knocked into the Avpr1b coding region to genetically target Avpr1b neurons. We confirmed this line has dense Cre expression throughout the dorsal and ventral CA2 regions of the hippocampus, along with scattered expression within the caudate-putamen and olfactory bulb (OB). Conditional removal of the NMDA receptor was achieved by crossing our line to an available floxed Grin1 line. The resulting mice were measured on a battery of social and memory behavioral tests. Surprisingly, we did not observe any differences between Avpr1b-Grin1 knockout mice and their wildtype siblings. We conclude that mice without typical NMDA receptor function in Avpr1b neurons can develop normal aggression as well as short-term social and object memory performance.

## Significance Statement

Activity of neurons that express vasopressin 1b receptor is essential for aggressive and social recognition behaviors. We created a novel transgenic mouse to allow selective targeting of vasopressin 1b neurons. Our studies indicate that NMDA receptor expression in vasopressin 1b neurons (including most CA2 neurons) are not required for the development of the typical expression of aggression or recognition memory. Thus, CA2 neurons may have a unique way of incorporating novel stimuli into memory that deserves further investigation.

## Introduction

The neuromodulatory actions of arginine-vasopressin (Avp) are required for the typical expression of social and stress-related behaviors in clinical populations and preclinical models (Caldwell et al., 2017; Williams Avram and Cymerblit-Sabba, 2017). Avp acts in the central nervous system through its G-protein coupled receptors, Avpr1a and Avpr1b. Recent data indicate that variation in Avpr1b signaling may have maladaptive impacts on human social behavior. Avpr1b single nucleotide polymorphism (SNP) variants are positively correlated with autism diagnoses (Francis et al., 2016) and with increased emotional aggression (Luppino et al., 2014). Similarly, life-long disruption of Avpr1b signaling through genetic removal in knockout (KO) mice results in altered social aggression and social memory performance (Wersinger et al., 2002), while leaving spatial and object memory performance and olfactory discrimination intact, indicating a special role in the social aspect of memory (Wersinger et al., 2002, 2004, 2007; Caldwell et al., 2008). Resident-intruder aggression in Avpr1b KO mice was decreased without co-occurring deficits in predatory aggression or anxiety and remained deficient in a KO on a more outbred strain of mice (Wersinger et al., 2007, 2008; Caldwell and Young, 2009; Caldwell et al., 2010). Furthermore, decreases in social aggression occurred in both sexes, suggesting a conserved signaling mechanism for males and females, unlike many of Avp’s actions through Avpr1a (Terranova et al., 2016; Williams Avram and Cymerblit-Sabba, 2017).

The anatomical specificity of the Avpr1b gene expression makes it a potentially desirable pharmacological target for translational studies investigating social memory or aggression, as there would be fewer possible behavioral side effects of systemic administration. Mapping of Avpr1b mRNA expression in mice revealed the highest levels in the pyramidal neurons of the dorsal cornu ammonis 2 (CA2) hippocampal region, with a few cells expressing in the medial amygdala and paraventricular nucleus of the hypothalamus (Young et al., 2006). Pagani et al. (2015) used lentiviral delivery of the Avpr1b gene into the dorsal CA2 of Avpr1b KO mice and were able to rescue the aggressive phenotype, indicating Avpr1b expression in the dorsal CA2 is essential to expression of species-typical aggressive behavior. Recently, Avpr1b activity in the dorsal lateral septum has been shown to regulate resident-intruder aggression through it actions on the presynaptic plasticity of dorsal CA2 neurons (Leroy et al., 2018). In addition to aggression, Avpr1b-expressing neurons in the dorsal hippocampus play an important role in social memory regulation. Optogenetic activation of vasopressinergic neuron terminals in the dorsal CA2 enhances and extends social memories (Smith et al., 2016). Local pharmacological antagonism of Avpr1b prevents this extended social memory, suggesting Avpr1b activity in the dorsal CA2 is required for the effect. However, the molecular mechanism through which Avpr1b may be influencing neuronal plasticity to contribute to these behavioral functions is unknown.

NMDA receptor signaling is required for plasticity in postsynaptic response in many cell types (Baez et al., 2018). The NMDA receptor is a tetrameric ion channel composed of two obligatory glutamate receptor ionotropic NMDA type 1 (Grin1) subunits and 2 subunits from the Grin2 or Grin3 families, the composition of which varies with cell-type and throughout development (Hansen et al., 2018). Genetic removal of Grin1 gene results in deficits in synaptic plasticity and learning (Nakazawa et al., 2004; Zweifel et al., 2008; Hansen et al., 2018). Avp application to dorsal CA2 neurons in mouse and rat *ex vivo* slices results in increased excitatory post-synaptic current (EPSC) amplitude lasting at least 15 min after Avp washout, a response that is blocked by NMDA glutamate receptor antagonists (Pagani et al., 2015). Furthermore, the increased EPSC amplitude was not mediated by altered local inhibitory signals as bicuculline had no effect on this response. This indicates that NMDA receptors may be critical for Avp’s role in modulating dorsal CA2 neural activity.

To determine the role that plasticity may have in Avpr1b neuron-dependent behaviors, we selectively inactivated NMDA receptors in Avpr1b neurons through genetic removal of the obligatory Grin1 gene, and assessed aggression, memory and anxiety-like behaviors. We predicted that these KO mice (Avpr1b^Grin1−/−^) would exhibit impairments in social memory performance and aggressive behaviors. Surprisingly, we did not observe any behavioral impact in any measure in the Avrp1b^Grin1^ mice, suggesting that a chronic loss of the Grin1 subunit in Avpr1b neurons is well tolerated with regard to social aggression and memory.

## Materials and Methods

### Generation of Avpr1b-Cre Knock-in Mice

**Figure 1:**
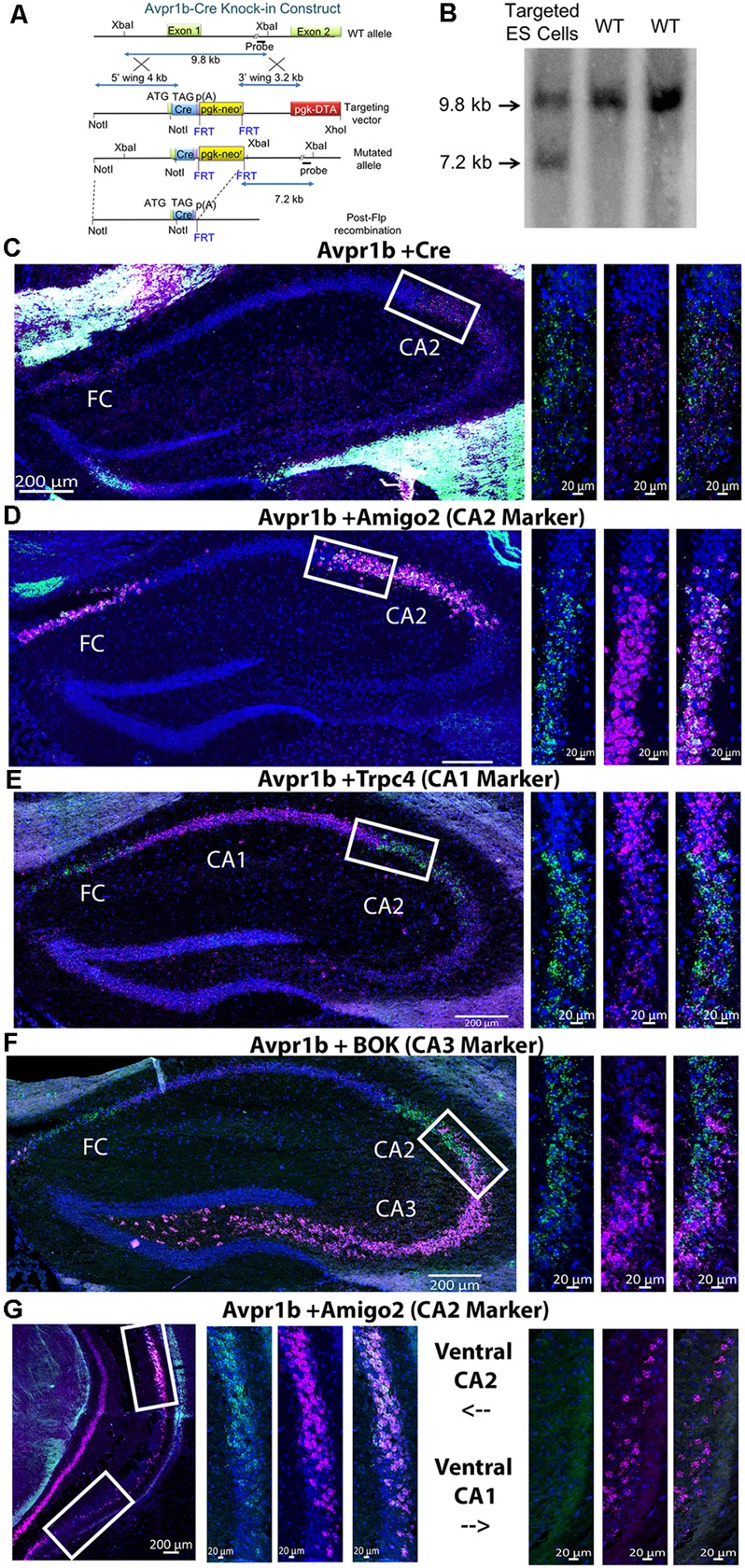
**(A)** An illustration of the design of the targeting vector construct indicating the portion of the arginine vasopressin 1b receptor (Avpr1b) gene surrounding exon 1 that was replaced through homologous recombination. **(B)** Southern blot indicating the Cre recombinase gene was knocked into the appropriate locus (genomic DNA restricted with XbaI). **(C)** Representative images of double *in situ* hybridization histochemistry on sections (16 μm) from a heterozygous mouse demonstrating Cre recombinase mRNA is selectively expressed in neurons that co-express Avpr1b mRNA in the dorsal hippocampus (*n* = 3 mice). Avpr1b transcripts locations are indicated by green dots and Cre by pink dots (bar = 200 μm). Representative magnified images of the white box show the border of the CA1 and cornu ammonis 2 (CA2; bar = 20 μm). **(D)** Representative images of double *in situ* hybridization histochemistry on sections from a wildtype (WT) mouse demonstrating the distribution of Avpr1b (green dots) and the CA2 marker, Amigo2 (pink dots), in the dorsal hippocampus (*n* = 2 mice). Representative magnified images of the white box show the border of the CA1 and CA2 (bar = 20 μm). **(E)** Representative images of double *in situ* hybridization histochemistry on sections from a WT mouse demonstrating the distribution of Avpr1b (green dots) and the CA1 marker, Trpc4 (pink dots), in the dorsal hippocampus (*n* = 2 mice). Representative magnified images of the white box show the border of the CA1 and CA2 (bar = 20 μm). **(F)** Representative images of double *in situ* hybridization histochemistry on sections from a WT mouse demonstrating the distribution of Avpr1b (green dots) and the CA3 marker, Bok (pink dots), in the dorsal hippocampus (*n* = 2 mice). Representative magnified images of the white box show the border of the CA2 and CA3 (bar = 20 μm). **(G)** Representative images of double *in situ* hybridization histochemistry on sections from a WT mouse demonstrating the distribution of Avpr1b (green dots) and the CA2 marker, Amigo2 (pink dots), in the ventral hippocampus (*n* = 2 mice). Representative magnified images of the upper white box show the border of the ventral CA2 (bar = 20 μm). Representative magnified images of the lower white box show the ventral CA1 (bar = 20 μm).

To generate the targeting vector construct (Figure 1A), a BAC plasmid clone (AC120217) that contains the mouse Avpr1b genomic locus was used to amplify various genomic segments by PCR. A 4 kb 5′ fragment containing the Avpr1b promoter and part of exon 1 with its start codon was inserted into a targeting vector [pGKneoF2L2 DTA (diphtheria toxin A gene)], kindly donated by Dr. Philippe Soriano at Fred Hutchinson Cancer Research Center, MIT, Boston, MA, USA) upstream of a phosphoglycerate kinase promoter-driven neomycin resistance cassette (pgk-neo^r^ or neo) flanked by FLP recombinase target (FRT) sites. The Cre-nuclear localization sequence (from the pBS185 plasmid from the former Life Technologies, Gaithersburg, MD, USA) and SV40 poly A signal (from the pTRE-tight plasmid from Clontech Laboratories, Mountain View, CA, USA) sequences were cloned right after the 5′ homology fragment. For the 3′ homology part of the construct, a 3.2 kb fragment containing intron 1 was inserted between the neo and pgk-DTA cassettes which were used to select against random insertion. Following electroporation into embryonic stem (ES) cells (LC3 cell line; Tra et al., 2011), DNA from G418 resistant clones was screened by PCR and digested with XbaI for analysis by Southern blotting using an internal probe. Correctly targeted alleles produced a 7.2 kb XbaI fragment owing to the insertion of the selectable drug-resistant maker (neo gene), compared to the wildtype (WT) allele of 9.8 kb (Figure 1B). The positive ES cells were injected into blastocysts to generate chimeras. Avpr1b-Cre heterozygous mice (Hets) were then crossed with a transgenic mouse expressing FLPe recombinase using a human beta-actin promoter [B6;SJLTg(ACTFLPe)9205Dym/J; The Jackson Laboratory, Bar Harbor, ME, USA] to remove the neo cassette that was flanked by FRT sites (Rodríguez et al., 2000). These Avpr1b-Cre heterozygotes (Avpr1b-Cre^+/−^) were then either bred together or with C57Bl/6J partners. Mice used in gene expression experiments were from litters backcrossed at least 3–4 generations into the C57Bl/6J strain.

Cre-dependent TdTomato reporter mice (Ai9) were purchased from Jackson Laboratory [Strain Name: B6.Cg-Gt(ROSA)26Sortm9(CAG-tdTomato)Hze/J, Stock Number: 007909]. These mice were bred to Avpr1b-Cre^+/−^ mice, resulting in Avpr1b-Cre^+/−^;TdTomato double transgenic offspring. Two adult males and females were used to assess Avpr1b-Cre distribution.

Mice containing loxP sites flanking the Grin1 (Grin1^lox/+^) gene were purchased from Jackson Laboratory (Strain Name: B6.129S4-Grin1^tm2Stl^/J, Stock Number: 005246; Tsien et al., 1996) and backcrossed 2–3 generations into the C57Bl/6J strain prior to breeding. Following this, heterozygotes were then bred together to maintain the colony. To generate experimental animals, male mice with a confirmed genotype of fully floxed Grin 1 and WT Avpr1b (Grin1^lox/lox^: Avpr1b^+/+^) were mated to females that were genotyped heterozygous floxed Grin1 and heterozygous for the Avpr1b-Cre (Grin1^lox/+^:Avpr1b^+/Cre^). The resulting offspring had the following genotypes: (1) Grin1^lox/lox^:Avpr1b^+/+^; (2) Grin1^lox/+^:Avpr1b^+/+^; (3) Grin1^lox/+^:Avpr1b^+/Cre^; and (4) Grin1^lox/lox^:Avpr1b^+/Cre^. The first two genotypes were labeled as WT. The third group were labeled as heterozygous Avpr1b^Grin1+/−^. The last group were labeled as the Grin1 KO Avpr1b^Grin1−/−^. Animals for behavioral experiments came from a total of six different breeding pairs.

Stimulus mice used in social recognition testing were sexually naïve, ovariectomized Balb/c adult females. They were purchased from Jackson Labs (Stock #000651) and ovariectomied. Briefly, the animal was anesthetized with ketamine/xylazine cocktail, and monitored until no pain response was noted. Then a small dorsal midline incision was made, the muscle wall spread using forceps, and the ovaries were removed. Following a 2-week recovery period, females were singly housed for at least 1 week prior to testing. Stimulus mice used in the social habituation and social novelty tasks were adult sexually-naïve group-housed C57BL/6J males bred in our colony. On an annual basis, new breeders are acquired from Jackson Labs (Stock #000664). Stimulus mice for the aggression testing were adult sexually-naïve group-housed Balb/c males (Stock #000651). Stimulus mouse choice for each test is based on extensive pilot work in our lab showing the strongest and most consistent response from group and single-housed male C57Bl/6J mice. Stimulus mice were used only once per day.

### Genotyping

Mice were genotyped by PCR using DNA extracted from tail snips. A single forward primer, V1BR#9 (GAAACGGCTACTCTCTCCGATTCCAAAAGAAAG), was designed for amplification of both WT and recombined loci. The first reverse primer, V1BR#5 (ACCTGTAGATATTTGACAGCCCGG), was designed to amplify the WT loci (762 bp PCR product). The second reverse primer, Cre.c35 (GATATAGAAGATAATCGCGAACATCTTCAGGTTCT), was designed to detect the Cre recombinase transgene (679 bp). PCR was carried out for 40 cycles with denaturation at 94°C, annealing at 60°C, and extension at 72°C, all for 1 min. Grin1 mice were genotyped using a forward primer NR1loxP2 (ACAATAGAGATTCAAGGCTGATCAAGG) and reverse primer NR1loxP3 (CTCTGGGTGGCTTGCCTGGCTGTATGTT). PCR was carried out for 40 cycles with denaturation at 94°C, annealing at 60°C, and extension at 72°C, all for 45 s. TdTomato mice were genotyped for the WT band using a forward primer, oIMR9020 (AAGGGAGCTGCAGTGGAGTA) and reverse primer, oIMR9021 (CCGAAAATCTGTGGGAAGTC). The mutant band was genotyped using the forward primer, oIMR9105 (CTGTTCCTGTACGGCATGG) and reverse primer, oIMR9103 (GGCATTAAAGCAGCGTATCC). PCR was carried out for 40 cycles with denaturation at 95°C, annealing at 65°C, and extension at 72°C, all for 45 s.

### RNA Chromogenic *in situ* Hybridization Histochemistry

For *in situ* hybridization histochemistry studies, mice were anesthetized with isoflurane, decapitated and the brains removed within 5 min and immediately frozen on powdered dry ice. Brains were sliced on a cryostat (3050S, Leica Microsystems) and 16 μm sections were collected onto slides and kept frozen until the time of assay. Avpr1b and Cre recombinase transcripts were simultaneously detected in fresh-frozen brain sections from heterozygous Avpr1b-Cre mice using the Affymetrix ViewRNA duplex kit (Catalog number: QVT0013; Santa Clara, CA, USA) as previously described (Young et al., 2018). Alternate slices were used to detect Grin1 and glutamic acid decarboxylase 1 (Gad1) expression. The probe sets targeted Avpr1b (catalog number: VB1-16867), Cre recombinase (VF6-16306), Grin1 (VB1-14161) and Gad1 (VB6-12632). A separate mouse was used for simultaneous detection of Avpr1b with the CA1, CA2, and CA3 markers. The CA1 marker used was the transient receptor potential channel 4 (Trpc4; VB1-2079), the CA2 marker was the adhesion molecule with Ig-Like domain 2 (Amigo2; VB6-19241) and the CA3 marker was BCl-2 related ovarian killer protein (Bok: VB1-3033354).

### Collection of Avpr1b-tdTomato Brains

Adult male and female Avpr1b-Cre^+/−^;TdTomato animals were transcardially perfused with 4% paraformaldehyde. Brains were removed and postfixed for 24 h. Following an overnight rinse in 1 M phosphate buffer saline solution, brains were transferred to a 30% sucrose solution. Brains were sliced at 50 μm on a cryostat (Leica3050) into cryoprotectant and mounted onto charged slides. Slides were stained with DAPI (300 nM) and coverslipped with polyvinyl alcohol/1,4-diazabicyclo[2.2.2]octane/glycerol solution (PVA-DABCO).

### Imaging and Image Analysis

All *in situ* hybridization sections were imaged using the Zeiss AxioScan Z1 slide scanner and online stitching and shading correction using a 20×, 0.8 NA objective. Image contrast was adjusted for presentation in Zen Blue. Image analysis was performed in Arivis Vision 4D software. For Avpr1b gene expression in the Avpr1b-Cre line, regions of interest were drawn over the dorsal CA2 and fasciola cinereum (FC). mRNA puncta were segmented based on intensity threshold and shape. Puncta were counted and presented as number per area. Measurements were made bilaterally on three sections/mouse. Measurements were then averaged per region and then averaged per mouse. For Grin1 gene expression, mean intensity of Grin1 label was quantified. Regions of interest were drawn using the nuclear counterstain (DAPI) channel. Regions were drawn around areas of strong Avpr1b expression including the CA2 and FC. Adjacent CA1, CA3, dentate gyrus and layer 2 motor cortex was measured. Measurements were made bilaterally on three sections/mouse. Measurements were then averaged per region, normalized to cortical measurements and averaged per animal. Three animals in each genotype were measured.

Images of cell body and fiber distribution of the Avpr1b-Cre^+/−^;TdTomato mice were collected using the Zeiss AxioScan Z1 slide scanner and online stitching and shading correction using a 20×, 0.8 NA objective. Image contrast was adjusted for presentation in Zen Blue. Images of the cell bodies in each anatomical location were collected on a Nikon C2 point-scanning confocal microscope using a 20×, 0.75 NA objective. Z-stacks were collected using a resolution of 1,024 × 1,024 pixels/field and 2× averaging. Z stacks then underwent 3D deconvolution in Nikon Elements software, using automatic settings and 15–25 iterations. Resulting images were further processed using “Rolling Ball” background correction with the radius set to 28 μm.

### Mouse Housing Conditions

All housing and procedures were approved by the Animal Care and Use Committee of the National Institute of Mental Health. Mice were housed in an AAALAC accredited specific pathogen-free vivarium kept at constant temperature and humidity (~21°C, 50%), in plastic micro-isolator cages (12″ × 6.5″ × 5.5″) containing wood chip bedding (Nepco Beta-chips) and cotton nestlets. All cages were maintained on high-density ventilated racks (Super Mouse 750, Lab Products Inc.). Mice were maintained on a 12-h light cycle (lights off at 1,500 h) with *ad libitum* access to standard mouse chow (Purina Lab Diet, Product #5R31) and water bottles. Cages were changed on a bi-weekly basis primarily by the same animal caretaker. All breeding pairs were fed a high-fat diet (Purina Lab Diet, Product #5058) to reduce pregnancy and pup loss. All offspring were weaned at ~21 days of age into cages with their same-sex littermates. No animals that were singly housed during adolescence were used in behavioral testing but may have been used for gene expression studies. All animals used in behavioral experiments were adult males that remained group-housed with littermates for the entirety of testing. Testing began when the animals were 90–120 days old.

### Behavioral Procedures

#### Experimental Design

All mice went through the same series of behavioral tests in the following order: Elevated O-Maze, Social Recognition Novel-Familiar, Social Recognition Novel-Novel, Social Habitation-Dishabituation, Object Habituation-Dishabituation, Olfactory Habituation-Dishabituation, Social Novelty Preference, and following a 2-week period three tests of aggression. For the elevated O-maze test, each mouse was measured sequentially, while the recognition, habituation, and aggression tasks were parallelized to allow four mice to be tested simultaneously. For simultaneous testing scenarios, all test spaces were assessed for even illumination. All behavioral testing occurred between 9:00 AM and 2:00 PM. All behavioral tests were separated by 48–72 h. Animals did not receive cage changes during the time between elevated maze testing and social novelty testing. Mice received fresh cages and remained undisturbed until aggression testing was completed. Three small groups of animals were run through the battery of tests. The groups were separated by several months, however, each group contained representatives from all genotypes. The total number of animals in each group was WT = 9, Avpr1b^Grin1+/−^ = 8, Avpr1b^Grin1−/−^ = 11. All testing was performed during the light phase of the cycle. All testing was performed in a room separated from the colony room.

#### Elevated O-Maze

Anxiety-like behavior was tested in an elevated O-maze (San Diego Instruments, San Diego, CA, USA). Testing took place in a darkened room (20 lux). A row of LED lights controlled by a dimmer switch was set to illuminate only the open arms at an intensity of 60 lux at the maze surface. All mice were moved into an anteroom of the behavioral suite 30 min prior to testing. Mice were placed on the open arm of the maze facing a closed arm and were allowed to explore the maze for 5 min. Mice were recorded using a ceiling-mounted camera connected to a Dell computer running Ethovision software (Noldus Information Technology, Leesburg, VA, USA). Trials began as soon as the experimenter left the room.

#### Social Recognition Testing

For the two-trial recognition test, mice were brought to the testing room and habituated to a fresh mouse cage containing clean bedding for 30 min. An unfamiliar ovariectomized Balb/C female was placed in the cage and allowed to freely interact with the experimental male for 5 min. Then the female was returned to her home cage. The experimental animal remained in the testing cage. Following a 30-min interval, the experimental mouse was re-exposed to either the original or a novel female for another 5-min interval.

#### Social Habituation-Dishabituation

Mice were brought to the testing room and habituated to fresh cages for 30 min. A group-housed adult C57Bl/6J stimulus male was added to the cage for four 1-min trials of unrestricted interaction with an inter-trial interval of 3 min. During the intervals, the stimulus mouse was kept in a holding container on an adjacent table and the experimental animal remained in the testing cage. The fifth trial introduced a novel stimulus male from a different home cage.

#### Object Habituation-Dishabituation

This procedure is similar to the social habituation protocol, except novel objects were placed in the cage. Objects were placed into the same corner of the cage for each trial. Location of the corner was randomized. The objects were small brightly colored plastic items with complex shapes (small water bottles, rubber duckies, scintillation vials with colored fluid). All items were cleaned with ethanol and water prior to use. All items were previously found to elicit similar amounts of initial investigation.

#### Olfactory Habituation-Dishabituation

Olfaction was tested using a habituation-dishabituation task adapted from one we have used previously (Lee et al., 2008). Mice were placed in a clean cage containing fresh bedding. Following a 30-min habituation, three odorants were presented for 1 min, three times each, with a 3-min inter-trial interval: water, almond (1:100), male mouse urine (1:100), for a total of nine trials. Presentation was made with 100 μl of odorant solution added to cotton balls placed in metal strainer spheres. The amount of time mice spent with their snout in proximity (1 cm or less) to the metal strainer was recorded. Odorant solutions were diluted in distilled water from stock solutions. Almond scents were extracts (McCormick and Company Inc., Spark, MD, USA) and urine samples were mixtures from several adult male mice. Urine was collected by placing animals in modified mouse metabolic chambers that have a grid mesh floor with a funnel to collect liquid below. Individual animals were allowed to stay in the chamber for 30 min. Urine samples were combined, flash-frozen on dry ice and stored at −80°C. Urine was thawed, diluted, and kept at 4°C between testing days.

#### Social Novelty Preference

Test mice were brought to the experimental room and allowed to acclimate for 30 min. Test mice were placed in the center of the Plexiglas three-chambered apparatus (each chamber 18 × 45 × 30 cm) for a 5-min habituation phase, followed by a 5-min choice phase. The side chambers contained either a male littermate who had been group-housed with the experimental mouse or a novel age- and weight-matched C57BL/6J male mouse. Stimulus mice were wearing collars and leashes that were affixed to the corner of the chambers. This prevented the stimulus mouse from leaving its designated chamber but allowed for full access to the stimulus mouse by the test animal (adapted from Winslow, 2003). The collars were small beaded plastic zip ties affixed to small 4-inch chains mounted on the wall of the chamber. The chains were mounted to the wall most distal to the entry to the chamber. The length of the tether allowed the stimulus mouse to access half of the chamber. Stimulus mice were habituated to the collars for 15–30 min prior to being placed in the chamber.

#### Aggression

Following 2 weeks of isolated housing, subjects were tested on three occasions, always 48–72 h between testing sessions. Subjects were brought to the testing room (~70 lux, with 60 dB white noise), between 9:00 AM–1:00 PM, weighed, and returned to their home cage without food or water for 30 min. Adult, weight-matched sexually-naïve, group-housed Balb/C male mice were used as intruders. Intruders were placed on the opposite side of the cage. Experimental males were given 5 min to exhibit aggressive behavior. If aggressive behavior was observed, the latency was noted, and the intruder removed after a 2 min period. Stimulus males were never used more than once on the same day and never with the same subject on a separate testing session. Intruder males were no longer used after they had been attacked five times. If an intruder male was observed initiating an attack on a subject, he was removed from the test and no longer used as an intruder. Videos were recorded with a Panasonic HDC-TM700 camera and scanned by an observer blind to the identity of the mouse for the initial instance of aggression defined as the first bite observed from the subject coded.

#### Behavioral Analysis

Videos of behavioral tests were recorded from above and coded by an observer blind to the identity of the mouse using JWatcher Software^1^ (Blumstein et al., 2010). The duration and frequency of all behaviors were recorded.

Behaviors analyzed for social behavior (aggression, social recognition, social habituation-dishabituation) tests included: (1) *Sniff anogenital*: the mouse sniffs the anogenital region of the stimulus, including the base of the tail. The mouse may approach from behind the stimulus mouse or burrow its head under the abdomen of the stimulus to place its nose between the hindlimbs. Sniffing may occur while mice are locomoting, where the mouse follows closely behind the stimulus, keeping its nose in contact with the anogenital region of the stimulus. (2) *Aggression*: the mouse attacks the stimulus mouse by lunging forward, biting, and often thrashing. When two mice are fighting, the mouse that initiated an attack is considered the aggressor. Attacks are commonly foreshadowed by circling (aggressor mouse runs circles around stimulus mouse with head oriented toward them) and/or tail rattling (aggressor mouse raises and rapidly shakes its tail while facing toward the stimulus mouse). (3) *Allogrooming*: the mouse licks the fur of another mouse (often rapidly) or uses its forepaws to comb the fur of another mouse. Grooming is usually focused on the back, shoulders and head of the recipient mouse, and the recipient mouse may either continue to behave freely or freeze and remain motionless while being groomed. (4) *Mounting*: the mouse approaches another mouse from the rear and places its forepaws on the back of the recipient mouse while thrusting its lower body forward. The recipient mouse will often attempt to flee, with the test mouse following closely behind and attempting to re-mount. General nonsocial behaviors were also measured. (5) *Autogrooming*: while sitting still, the mouse licks its fur, swipes its face with its forepaws, and/or uses any limb (though usually hindlimbs) to scratch itself. (6) *Digging/burrowing*: the mouse uses its face or limbs to actively displace bedding. The mouse may bury its face in bedding while sitting still or push bedding in front of it while moving using its head and forepaws. (7) *Climbing*: the mouse moves to any surface other than the floor of the cage or testing chamber. Climbing may occur on the lip of the cage or testing chamber, on the metal air vent attached to the cage, or on top of a corral or other object within the cage. The mouse may be still or in motion while climbing. (8) *Rearing*: the mouse raises its forepaws off of the floor and sits or stands on its hindlimbs. The mouse may sit on its haunches with its forepaws in the air, or stretch with its hindlimbs extended and its forepaws resting on an object or on the side of the cage or testing chamber.

Behaviors analyzed for object and olfactory habituation experiments included: *Sniff*: the mouse sniffs the object, indicated by head movement and snout within 1 cm of object. Sniffing may occur while mice are walking. *Climbing, Autogrooming, Digging/burrowing, and* *Rearing* are scored as described above.

Behaviors analyzed for the elevated O-maze included: (1) *light/dark arm entries*: the mouse must have all four paws within the boundary. (2) *Entering into light/dark arm*: mouse remains within the first 10 cm of the arm, facing into the arm. (3) *Stretch into Arm*: while in the open arm, the mouse leans forward and stretches its forepaws into the opposite arm. The hindlimbs must remain in the original arm. (4) *Head-dipping*: the mouse stopped walking and peered over the side of the open arm (5) *Rearing* was scored as described above. (6) *Light/dark arm duration*: the total amount of time spent in each portion of the maze. Very little head-dipping or rearing were observed, so these times were collapsed with the total arm duration. (7) *Distance Traveled*: Ethovision software provided the total distance traveled as a measure of locomotor activity.

### Statistical Analysis

All data were checked for consistency with normality using the Shapiro–Wilks test. Avpr1b gene expression was assessed with a student’s *t*-test. Grin1 mRNA expression was assessed with two-way ANOVA and planned *post hoc* comparisons. Litter number comparisons used the Kolmogorov–Smirnov test. The elevated O-maze measures were analyzed using one-way ANOVAs and Welch’s test (for non-normal) to compare genotypes. All memory and habituation behavioral measures were analyzed using two-way ANOVAs comparing Genotype as a between-group factor and treating Trial as a repeated measure within-group factor, with planned comparisons of genotype in Trial One. Additionally, ratio scores were computed and compared with one-way ANOVAs or Kruskal–Wallis (for non-normal sets). Aggression likelihood test used a Chi-square analysis to compare genotypes. Aggression duration and frequency were compared with two-way repeated measures ANOVAs with Geissner-Greenhouse correction for unequal variance. All data analysis was performed in Prism Software (version 8).

## Results

### Validation of Transgenic Mouse Model

#### Normal Growth and Development in Avpr1b-Cre Animals

The transgenic knockin did not result in any abnormal breeding occurrences. Specifically, the genotypic ratio was not different than expected (expected; WT = 50%, Avpr1b-Cre = 50%; Born: WT = 45%, Avpr1b-Cre = 55%, *χ*^2^, *df* = 0.50, 1, *p* = 0.5). Litters size was not significantly different than C57’s in our colony (Mean = 6.7 ± 2.1 vs. 6.03 ± 2.4, Kolmogorov–Smirnov = 0.18, *p* = 0.1). Furthermore, the adult weights of Avpr1b-Cre males did not significantly differ from WT (Avpr1b: 32.3 ± 3.3, WT: 29.9 ± 2.2; *t*, *df* = 1.9, 22, *p* = 0.06).

#### Cre Expression Matches Avpr1b Expression

Cre recombinase expression was confined to the same cells as Avpr1b gene expression as shown by *in situ* hybridization histochemistry. Avpr1b mRNA and Cre mRNA were seen in dorsal CA2 and immediately adjacent CA3 areas of the hippocampus (Figure 1C), consistent with our previous Avpr1b localization (Young et al., 2006). To confirm that the Avpr1b gene expression is restricted to the pyramidal cells of the CA2, double *in situ* hybridization was performed with the dorsal CA2 marker Amigo2 (Figure 1D). These images clearly show colocalization of Avpr1b gene with Amigo2, although the abundance of Amigo is much greater. This colocalization remains until the border of dorsal and ventral CA2 neurons (Figure 1G). Avpr1b gene expression was then observed in many fewer cells, while Amigo2 was observed in the most ventral regions of the CA1 and in posterior CA3 cells (Figure 1G). Avpr1b gene expression colocalization with a CA1 marker, Trpc4, was also investigated. In the dorsal hippocampus, there was a clear distinction between the cell types (Figure 1E). Trpc4 expression was noticeably greater in the CA1 compared to adjacent CA2 and CA3 subfields, while Avpr1b gene expression did not cross the CA1 boundary. In contrast, the border between the Avpr1b gene expression and that of the CA3 marker, Bok, appeared less distinct (Figure 1F). Nonetheless, there was minimal cellular colocalization of the Avpr1 with CA1 or CA3 markers in the dorsal hippocampus.

#### Avpr1b-Expressing Neuron Localization

**Figure 2:**
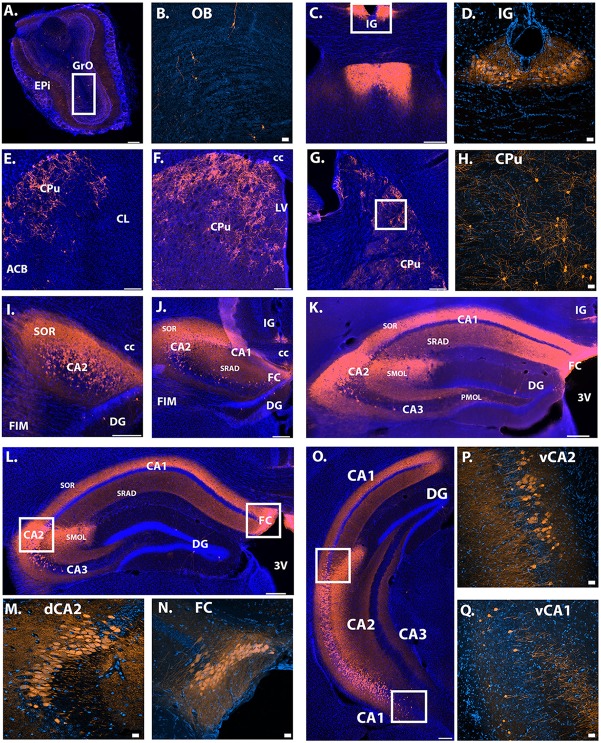
Location of Avpr1b-Cre throughout the adult brain using Cre-dependent TdTomato expression. **(A)** Scattered cells were observed in the main OB, found in both the GrO and Epi (bar = 200 μm). **(B)** Representative magnified image of the white box in **(A)** show the morphology of olfactory neurons. **(C)** Indusium Griseum neurons were observed quite anterior and continued posterior until the middle of the hippocampus **(K)**. **(D)** Representative magnified image of the white box in **(C)** shows the morphology of IG neurons. **(E–G)** Cells were observed throughout the anterior-posterior sections of the caudate putamen. **(H)** Representative magnified image of the white box in **(G)** shows the morphology of CPu neurons. **(I–L)** Densely packed neuronal cells were observed throughout the anterior-posterior dorsal CA2 and FC. **(M)** Representative magnified image of the left white box in **(L)** shows the morphology of dorsal CA2 neurons. **(N)** Representative magnified image of the right white box in **(L)** shows the morphology of dorsal FC neurons. **(O)** Neuronal cells were observed in the ventral CA2 and even into some ventral CA1 neurons. **(P)** Representative magnified image of the top white box in **(O)** shows the morphology of ventral CA2 neurons. Cells were found in the deep and superficial layers. **(Q)** Representative magnified image of the lower white box in **(O)** show the morphology of ventral CA1 neurons. Cells were found in the deep and superficial layers. Neuronal projections were observed within the dorsal hippocampus. Dense fibers were observed in the stratum oriens and stratum radiatum and dense dendritic projections were observed with the stratum lacunosum-moleculare of the CA2 in all layers where cells bodies exist **(I–O)**. Images collected with confocal displayed with the lighter orange color. Thick scale bars = 20 μm. Thin bars = 200 μm. GrO, granular olfactory layer; Epi, external plexiform layer; OB, main olfactory bulb; CC, corpus callosum; LV, lateral ventricle; 3V, third ventricle; FIM, fimbria; IG, induseum griseum; CPu, caudate putamen; ACB, nucleus accumbens; CL, claustrum; CA, cornu ammonis; SOR, stratum oriens; DG, dentate gyrus; SRAD, stratum radiatum; FC, fasiola cinereum; SMOL, stratum lacunosum-moleculare; PMOL, polymorphic layer of dentate gyrus; Blue cells, DAPI; Orange, Cre-dependent TdTomato.

**Figure 3:**
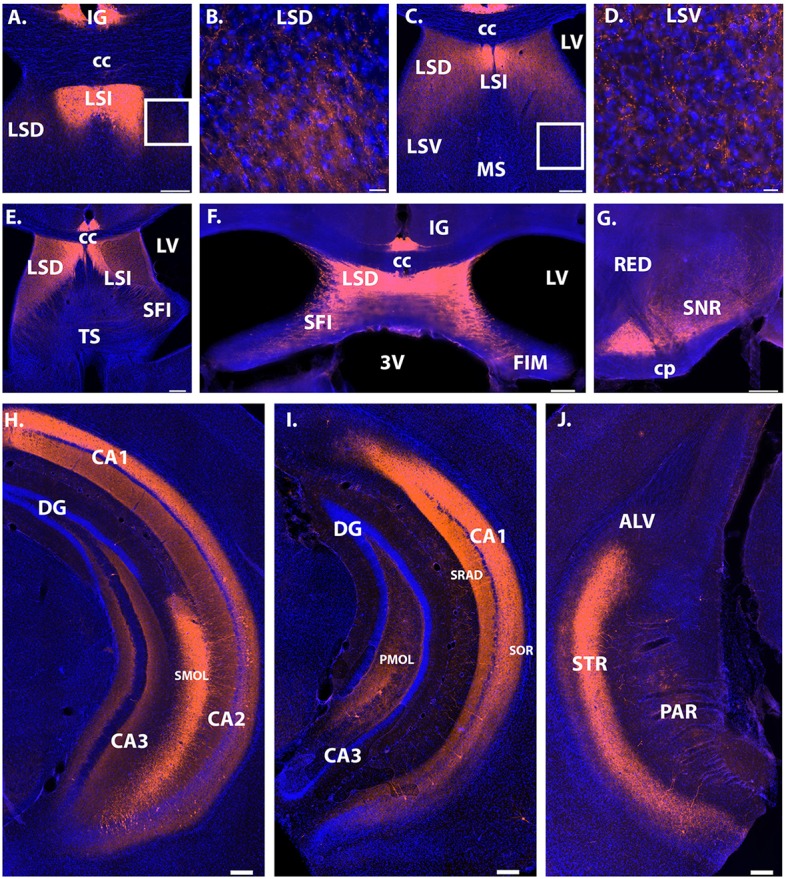
Projection sites of Avpr1b-TdTomato expressing neurons. Extra hippocampal fibers were observed throughout the lateral septum **(A–F)**. The densest projections were found in the LSD and LSI depending on anterior-posterior coordinate. **(A)** Very dense fibers in the LSI. **(B)** Representative magnified image of the white box in **(A)** shows the morphology of less dense fibers in the LSD. **(C)** Dense fibers in the LSI and LSD. No fibers observed in the MS. **(D)** Representative magnified image of the white box in **(C)** shows the morphology of fibers in the LSV. **(E)** Dense fibers in the LSI and LSD, with noticeable fibers in the SFI and TS. **(F)** Dense fibers in the LSD, with moderate density in the SFI. **(G)** Dense fibers in the ventral SNR, with moderate fiber density in the dorsal SNR. Dense fiber projections continued posteriorly throughout the posterior hippocampus and into the transition zone of the subiculum **(H–J)**. Dense fibers were observed in the stratum oriens and stratum radiatum of posterior CA1 **(H,I)**. Dense dendritic projections were observed within the stratum lacunosum-moleculare of the CA2 in all layers where cells bodies exist **(H)**. In posterior sections dense fibers were observed within the polymorphic layer of the dentate gyrus **(I)**. Scale bars = 200 μm except for **(B)** and **(D)** which are 20 μm. CC, corpus callosum; LV, lateral ventricle; 3V, third ventricle; FIM, fimbria; SFI, septofimbrial nucleus; LSD, lateral septum dorsal division; IG, induseum griseum; CA, cornu ammonis; SOR, stratum oriens; DG, dentate gyrus; SRAD, stratum radiatum; SMOL, stratum lacunosum-moleculare; PMOL, polymorphic layer of dentate gyrus; LSI, lateral septum intermediate; LSV, lateral septum ventral division; MS, medial septum; TS, triangular septal nucleus; ALV, alveus of the hippocampus; STR, transition zone of subiculum; PAR, parasubiculum; SNR, substantia nigra reticulata; CP, cerebral peduncle; RED, red nucleus. Blue cells, DAPI; Orange, Cre-dependent TdTomato.

To further characterize the extent of Avpr1b gene expression, the Avpr1b-Cre line was crossed with a fluorescent reporter line (male and female, *n* = 3). We saw cellular expression in the expected dorsal CA2 and FC neurons (Figures 2I–N), where the majority of the pyramidal cells were labeled. Additionally, we observed labeled neurons in ventral hippocampal areas (Figures 2O–Q). Notably, we observed many labeled neurons in areas that were typically described as ventral CA1 (Figures 2O,Q, 3I). A scattering of neurons was observed within the granule layer of dorsal and ventral dentate gyrus. Neurons of the induseum griseum appeared above the corpus callosum for an extended range (~1 mm; Figures 2C,D,J,K). Furthermore, we consistently observed numerous scattered cells throughout the caudate-putamen (Figures 2E–H). A few scattered cells were observed within the olfactory bulbs (OB) within the granular cell layer and the external plexiform layer (Figures 2A,B).

Intrahippocampal projections were observed within the dorsal and ventral hippocampus. Specifically, in the dorsal CA1, CA2 and CA3, fibers were most dense in the stratum oriens, with strong projections within the stratum radiatum (Figures 2I–L, 3H,I). Dendritic projections were observed in the molecular layer of CA2 and CA1 (2J–M,O). Projections within the molecular layer of CA1 continued past the location of cell bodies into the ventral hippocampus and persisted to the most posterior region (Figures 3H,I). Moderate projection density was observed in the polymorphic layer of the dentate gyrus dorsally and denser projections observed more ventrally (Figure 3I). Projections continued posteriorly into the transition zone of the subiculum (Figure 3J).

Extrahippocampal projections were limited to the septum and substantia nigra. Dense fiber projections were observed in the dorsal lateral septum and extending into the septohippocampal nucleus and intermediate lateral septum (Figures 3A–F). Less dense projections were observed in the ventral division of the lateral septum and the triangular nucleus of the septum. Projections were also observed throughout the substantia nigra, with the densest projections observed within the ventral part (Figure 3G).

#### Avpr1b Gene Expression Developmental Timeline

**Figure 4:**
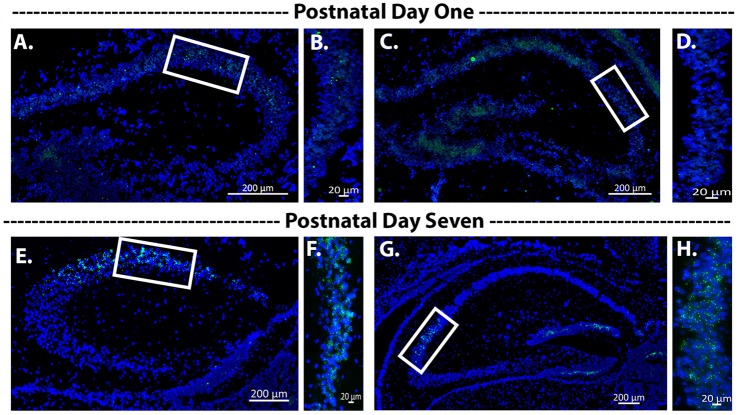
Avpr1b expression during early postnatal development. Representative images of *in situ* hybridization histochemistry on sections (16 μm) from a heterozygous mouse (*n* = 2). **(A–D)** Dorsal hippocampus sections from postnatal day 1 mouse brain along the rostrocaudal axis. Very sparse labeling is observed. **(B,D)** Representative magnified images of the white boxes in **(A,C)**, respectively. **(E–H)** Dorsal hippocampus sections form postnatal day 7 mouse brain along the rostrocaudal axis. Robust Avpr1b mRNA expression is seen across the CA2 and FC regions. **(F,H)** Representative magnified images of the white boxes in **(E,G)**, respectively. Blue, DAPI; Green, Avpr1b.

To determine when the Cre recombinase became active during development, we assessed Avpr1b gene expression at postnatal day 1 and postnatal day 7. We observed extremely limited expression within the hippocampus at postnatal day 1 (Figures 4A–D). However, by postnatal day 7 there was abundant expression within the dorsal and ventral hippocampus (Figures 4E–H).

#### Avpr1b Gene Expression in Avpr1b-Cre Animals

**Figure 5:**
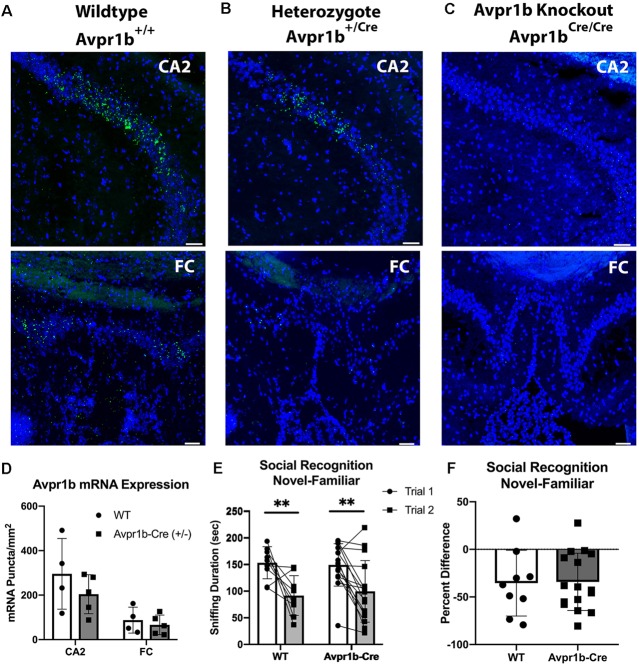
Cre insertion does not alter Avpr1b expression or social recognition behavior. Representative images of *in situ* hybridization histochemistry of Avpr1b in the CA2 and FC in WT animals **(A)**, Heterozygotes **(B)**, and Avpr1b-Cre knockouts (KO; **C**). These animals have two alleles of the Cre-recombinase after the Avpr1b promoter, and thus have no Avpr1b gene expression. Scale bar = 50 μm. **(D)** Quantification of number of puncta/area of Avpr1b gene in WTs and heterozygotes. Two-way ANOVA (Genotype × Region) revealed a significant effect of region (*F*_(1,7)_ = 44, *p* < 0.0001), but no effect of genotype (*F*_(1,7)_ = 0.9, *p* = 0.4). **(E)** Social interaction and Social Memory are intact in heterozygotes. Sniff duration is shown in the graph. A two-way repeated measures ANOVA revealed a main effect of Trial (*F*_(1,23)_ = 25.6, *p* < 0.0001), but no effect of genotype. **Indicates difference between Trial 1 and Trial 2 (*p* < 0.005). **(F)** Ratio of Trial 2 to Trial 1 sniffing duration. All data presented as individual data points overlaid on means ± SD.

To determine if Avpr1b mRNA expression is impacted by the Cre knockin in the dorsal hippocampus, Avpr1b gene expression was measured in the CA2 and FC in WTs (*n* = 4) and Avpr1b-Cre heterozygotes (*n* = 5; Figures 5A,B). The puncta/area were measured (Figure 5D) and two-way ANOVA (Genotype × Region) revealed a significant effect of region (*F*_(1,7)_ = 44, *p* < 0.0001), but no effect of genotype (*F*_(1,7)_ = 0.9, *p* = 0.4). No detectable Avpr1b was detected in mice homozygous for the Avpr1b-Cre knockin gene (Figure 5C).

#### Avpr1b-Cre Knock-in Does Not Alter Social Recognition Behavior

To assess whether the integration of Cre-recombinase into the Avpr1b locus resulted in behavioral changes, adult male Avpr1b-Cre heterozygous animals were compared with WT littermates on the social recognition test (Figures 5E,F). A two-way repeated measures ANOVA revealed a main effect of Trial (*F*_(1,23)_ = 25.6, *p* < 0.0001), but no effect of genotype (*F*_(1,23)_ = 0.01, *p* = 0.9). A planned *post hoc* comparison of Trial 1 sniffing showed no significant difference in the amount of initial investigation (Sidak’s multiple comparison adjusted *p* = 0.9). The ratio scores also did not differ between the genotypes (*t*_(2)_ = 0.10, *p* = 0.9).

### Validation of the Avpr1b^Grin1+/−^ Transgenic Line

#### Normal Growth and Development in Avpr1b^Grin1−/−^ Animals

The transgenic cross did not result in any abnormal breeding occurrences and the ratio of the three genotypes was as expected given our breeding strategy (expected: WT: 50%, heterozygotes: 25%, KOs 25%). Specifically, Avpr1b^Grin1−/−^ males were 8%, Avpr1b^Grin1−/−^ female were 10%, Avpr1b^Grin1+/−^ males were 13%, Avpr1b^Grin1+/−^ females were 16%, WT males were 28% and WT females were 25%, compared to expected (*χ*^2^, *df* = 1.15, *p* = 0.5). Litters were normally sized (6.9 ± 2.3) and did not significantly differ in size compared to the Avpr1b-Cre line (Kolmogorov–Smirnov = 0.04, *p* = 0.99).

#### Grin1 Is Removed From Pyramidal Cells in the Dorsal CA2

**Figure 6:**
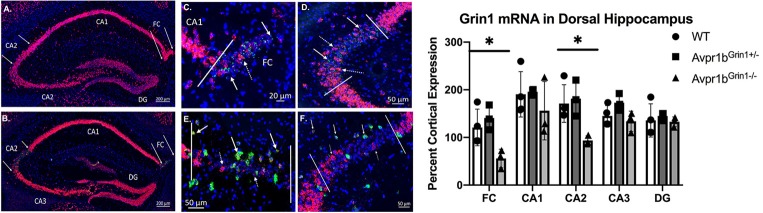
Grin1 is reduced in transgenic mice. Double *in situ* hybridization of Avpr1b (green) and Grin1 (red) mRNA in the dorsal hippocampus in WT **(A)** and Avpr1b^Grin1−/−^ **(B–F)**. Areas of Avpr1b gene expression are located between the solid arrows in **(A,B)**. **(C,D)** Magnifications of FC **(C)** and CA2 **(D)** from **(B)**. Solid lines indicate boundaries of Avpr1b gene expression. Solid arrows indicate cells with Avpr1b expression and no Grin1. Dotted arrows indicate cells with remaining Grin1 within the region. **(E,F)** Double *in situ* hybridization of Grin1 (red) and glutamic acid decarboxylase (GAD; green). Solid lines indicate boundaries of the FC **(E)** and CA2 **(F)**. Solid arrows indicate GAD positive cells with Grin1 expression within these regions. Dotted arrows indicated GAD negative cells with Grin1 expression. **(G)** Quantification of Grin1 intensity in WT, Avpr1b^Grin1+/−^, and Avpr1b^Grin1−/−^ mice. Avpr1b^Grin1−/−^ mice had a significant reduction in Grin1 within the FC and CA2. *Indicate *p* < 0.01 compared to WT and Avpr1b^Grin1+/−^. Data presented as individual data points overlaid on means and SD.

Grin1 mRNA was quantified throughout the dorsal hippocampus where the expression of Cre was most prominent and localized (Figure 6). A two-way ANOVA comparing region and genotype revealed a main effect of region (*F*_(4,35)_ = 7.0, *p* = 0.003), and a main effect of genotype (*F*_(2,35)_ = 11.1, *p* = 0.0002). *Post hoc* Tukey’s tests revealed that Avpr1b^Grin1−/−^ mice were significantly different from Avpr1b^Grin1+/−^ (*p* = 0.005) and WT (*p* = 0.008) groups in the CA2. Avpr1b^Grin1−/−^ mice were significantly different from Avpr1b^Grin1+/−^ (*p* = 0.007) and WT (*p* = 0.03) groups in the FC. No differences were observed in the CA1, CA3 or dentate gyrus. We confirmed the removal of Grin1 from only pyramidal cells within CA2 and FC regions, as Grin1 was still colocalized with Gad mRNA expression (Figures 6E,F).

### Behavioral Results From Avpr1b^Grin1−/−^

#### Resident Intruder Aggression

**Figure 7:**
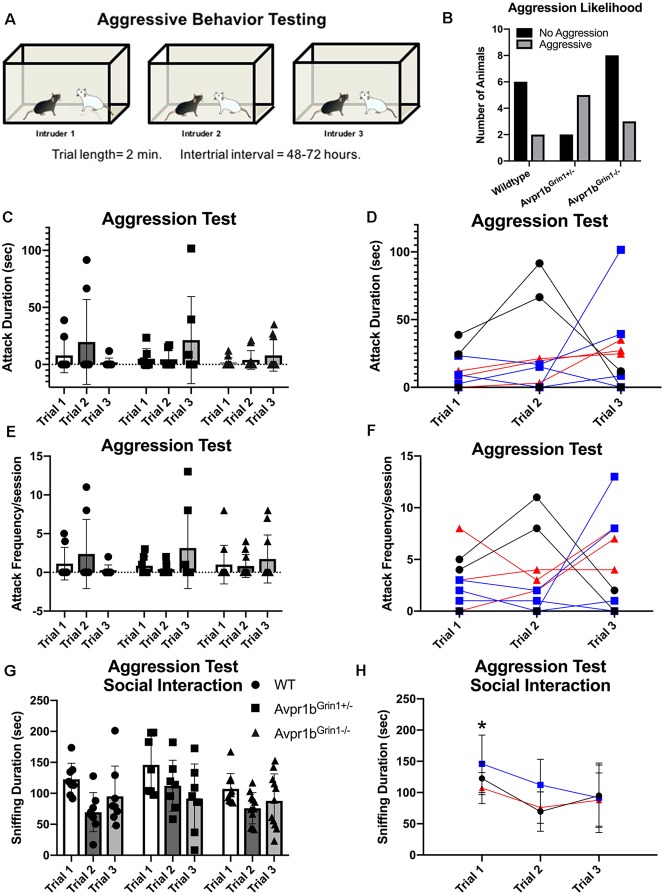
Aggressive behavior in Avpr1b^Grin1−/−^ mice. **(A)** Depiction of experimental design. Each mouse was tested on three occasions separated by 48–72 h, each time with a novel intruder. **(B)** Likelihood to attack. The number of mice that exhibited any aggressive behavior across the three sessions (black bars) and those that never exhibited any aggressive behaviors (gray bars). No significant difference in ratio was observed. **(C−F)** Aggression behaviors. Aggression duration **(C)** and frequency **(E)** data presented as individual data points overlaid on means ± SD. **(D,F)** Individual animals tracked across sessions. WTs are represented as black circles and lines. Avpr1b^Grin1+/−^ are represented as blue boxes and lines. Avpr1b^Grin1−/−^ are represented as red triangles and lines. **(G,H)** Sniffing behavior during the aggression test. **(G)** All data presented as individual data points overlaid on means ± SD. **(H)** Grouped data to illustrate the effect of trial. Two-way ANOVA (Trial × Genotype) revealed a main effect of Trial (*F*_(1,42)_ = 7.49, *p* = 0.002). *Post hoc* Tukey’s multiple comparisons revealed Trial 1 sniffing duration was significantly higher than all other trials. *p < 0.05 Trial 1 vs. Trial 2 and 3.

Experimental mice were tested using the resident-intruder paradigm once a day on three non-consecutive days (Figure 7A). There was no difference in the likelihood to attack an intruder (*χ*^2^ = 4.4, *df* = 2, *p* = 0.1; Figure 7B). A two-way repeated measures ANOVA indicated no difference in the duration of aggressive behaviors across trials (*F*_(1.3,30.6)_ = 0.83, *p* = 0.4) or across genotypes (*F*_(2,23)_ = 0.5, *p* = 0.9). A trend for an interaction (*F*_(4,46)_ = 2.4, *p* = 0.058), was observed; however, *post hoc* testing revealed no significant differences between the genotypes on any testing day or within a genotype across test days (Figures 7C,D). Similarly, two-way repeated measures ANOVA indicated no differences in the frequency of aggressive behaviors across trials (*F*_(1.3,31.5)_ = 0.7, *p* = 0.4) or across genotypes (*F*_(2,23)_ = 0.04, *p* = 0.9; Figures 7E,F). Non-aggressive social interaction toward the intruder was not significantly different between the groups, although a trend for reduced interaction was observed (*F*_(2,23)_ = 3.3, *p* = 0.057). All groups showed a habituation to sniffing the intruder over multiple testing sessions (Figures 7G,H). Two-way repeated measures ANOVA (Trial × Genotype) revealed a significant effect of Trial (*F*_(1,42)_ = 7.49, *p* = 0.002). *Post hoc* Tukey’s multiple comparisons revealed Trial 1 sniffing duration was significantly higher than all other trials [compared with Trial 2 (*p* = 0.001), Trial 3 (*p* = 0.04)].

### Social Memory Testing

#### Social Recognition: Novel-Familiar

**Figure 8:**
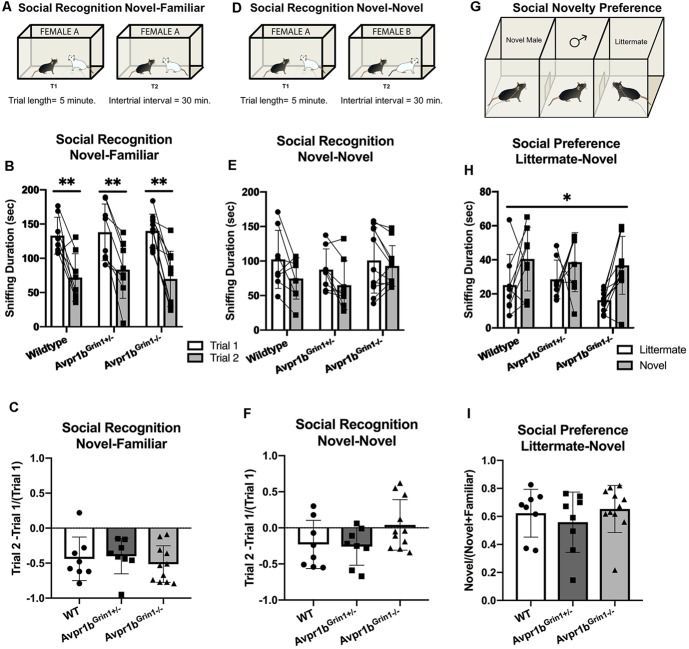
Social memory is unaffected by removal of NMDA receptor from Avpr1b neurons. **(A–C)** Short term (30 min) social recognition measured by repeatedly exposing the subject to the same stimulus animal across two trials (Novel-Familiar). **(A)** Graphic demonstrating the social recognition Novel-Familiar test. **(B)** Sniffing duration for Trials 1 and 2. Two-way ANOVA showed a main effect of trial (*F*_(1,24)_ = 75.7, *p* < 0.0001). *Post hoc* tests showed that within each genotype there was a significant difference between the two trials. **p < 0.01. **(C)** Ratio of Change between Trial 1 and Trial 2. One-way ANOVA revealed no differences. **(D)** Graphic demonstrating the social recognition Novel-Novel test. **(E)** Sniffing duration for Trials 1 and 2. Non-specific recognition response is measured by exposing the subject to different stimulus animals across the two trials (Novel-Novel). Two-way ANOVA showed a main effect of Trial (*F*_(1,24)_ = 10.7, *p* = 0.003). *Post hoc* comparisons confirm that no group had a significant difference between trials. **(F)** Ratio of change between Trial 1 and Trial 2. One-way ANOVA revealed no differences. **(G)** Social novelty preference compares interaction with a littermate and an age- and sex-matched novel animal when presented simultaneously in a 3-chamberd cage. **(H)** Sniffing duration for each stimulus mouse. Repeated measures ANOVA revealed a significant main effect of stimulus mouse (*F*_(1,20)_ = 11.1, *p = 0.003), but no effect of genotype. **(I)** Ratio of novel sniffing to total sniffing. One-way ANOVA revealed no differences. All data are presented as individual responses, with means ± SD overlaid.

No differences were observed in social recognition testing (Figures 8A–C). Two-way repeated measures ANOVA (Genotype × Trial) revealed a main effect of Trial (*F*_(1,24)_ = 75.7, *p* < 0.0001), with no effect of genotype (*p* = 0.9) on sniffing duration in the social recognition novel-familiar test. Avpr1b^Grin1−/−^ showed similar amounts of sniffing duration in the initial investigation of the stimulus mouse (Sidak’s multiple comparison, WT vs. Avpr1b^Grin1−/−^, *p* = 0.9, Avpr1b^Grin1−/−^ vs. Avpr1b^Grin1+/−^, *p* = 0.9). A ratio score was calculated to control for individual variability in initial investigation in Trial 1 (Trial 2 − Trial 1/Trial 1). A one-way ANOVA revealed no significant difference between the genotypes (*F*_(2,24)_ = 0.4, *p* = 0.6). Sniffing frequency was unaffected by genotype (*F*_(2,24)_ = 1.4, *p* = 0.25) or trial (*F*_(1,24)_ = 3.1, *p* = 0.09).

#### Social Recognition: Novel-Novel

To determine whether the reduction in Trial 2 was specific to the stimulus mouse, the same protocol was repeated but a novel mouse was presented in the second trial (Figures 8D–F). We saw no differences between the groups (*F*_(2,24)_ = 0.8, *p* = 0.4), however there was a main effect of Trial (*F*_(1,24)_ = 10.7, *p* = 0.003). *Post hoc* comparisons confirmed that no group had a significant difference between trials. A one-way ANOVA of the ratio scores revealed no significant differences between the genotypes in performance (*F*_(2,24)_ = 2.6, *p* = 0.09). Furthermore, there were no differences observed in the initial investigation in Trial 1 (*F*_(2,24)_ = 0.3, *p* = 0.72).

#### Social Novelty Preference

The social novelty preference design measures the subject’s choice to investigate a novel animal more in the presence of a very familiar animal, in this case, a male sibling with which they have continuously been housed. The stimulus mouse was an age-, weight- and sex-matched group-housed animal from the same strain (Figures 8G–I). For the sniffing behavior, a repeated measures ANOVA (Genotype × Novelty) revealed a main effect of Novelty (*F*_(1,24)_ = 12.2, *p* = 0.002). A ratio score for the investigation time was calculated to determine preference for the novel animal over the littermate [Novel Animal/(Novel Animal + Littermate)]. There was no significant difference between the genotype in the ratio score (Kruskal–Wallis = 1.03, *p* = 0.59). Furthermore, there was no difference in the total amount of investigation (Kruskal–Wallis = 2.5, *p* = 0.28). The total time spent in the side of the chamber yielded similar results. There was a main effect of novelty (*F*_(1,24)_ = 6.4, *p* = 0.01), with no effect of genotype (*F*_(2,24)_ = 0.7, *p* = 0.5). The ratio score did not differ based on genotype (Kruskal–Wallis = 0.35, *p* = 0.8).

#### Social Habituation-Dishabituation

**Figure 9:**
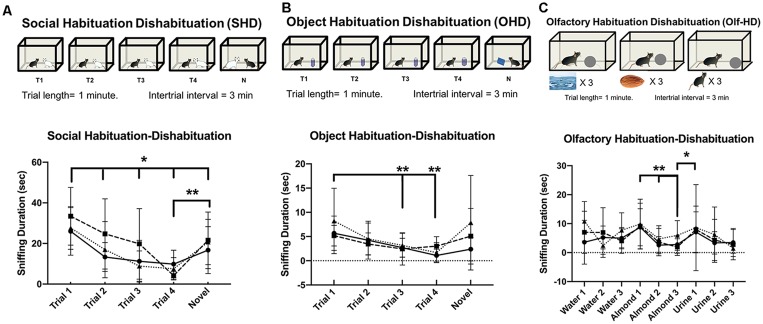
Habituation to social and non-social cues is unaffected by removal of NMDA receptor from Avpr1b neurons. **(A)** Social Habituation-Dishabituation. Graphic depicting the behavioral test (top). Sniffing duration across all trials (bottom). Two-way repeated measures ANOVA (Trial × Genotype) revealed a significant effect of Trial (*F*_(4,96)_ = 20.89, *p* < 0.001). *Post hoc* Tukey’s multiple comparisons revealed Trial 1 sniffing duration was significantly higher than all other trials, *p < 0.05. A robust dishabituation response was observed upon the presentation of the novel stimulus mouse revealed by comparing Trial 4 and the Novel Trial, **p < 0.001. **(B)** Object Habituation-Dishabituation. Graphic depicting the behavioral test (top). Sniffing duration across all trials (bottom). Two-way repeated measures ANOVA (Trial × Genotype) revealed a significant effect of Trial (*F*_(4,96)_ = 7.2, *p* < 0.001). *Post hoc* Tukey’s multiple comparisons revealed Trial 1 sniffing different from Trial 3 and 4, **p < 0.001. A robust dishabituation response was observed upon the presentation of the novel stimulus mouse revealed by comparing Trial 4 and the Novel Trial, *p < 0.002. **(C)** Olfactory Habituation-Dishabituation. Graphic depicting the behavioral test (top). Sniffing duration across all trials (bottom). Two-way repeated measures ANOVA (Trial × Genotype) revealed a significant effect of Trial (*F*_(8,192)_ = 6.1, *p* < 0.001). *Post hoc* Tukey’s multiple comparisons revealed significant decreases between the initial almond presentation and subsequent two trials (**p = 0.001).

The novel animal trial of social habituation-dishabituation test measures the ability of the subject to recognize an individual after a short exposure and short interval as well as discriminate between individual stimulus animals presented independently (Figure 9A). All groups performed similarly in the social habituation-dishabituation test. This task presented a new kind of stimulus animal (intact males from the same species). Two-way repeated measures ANOVA (Trial × Genotype) revealed a significant effect of Trial (*F*_(3.0,72.2)_ = 21, *p* < 0.001), but not genotype (*F*_(2,24)_ = 1.1, *p* = 0.4). *Post hoc* Tukey’s multiple comparisons revealed Trial 1 sniffing duration was significantly higher than all other trials [compared with Trial 2 (*p* = 0.005), Trial 3 (*p* < 0.001), Trial 4 (*p* < 0.001), Novel (*p* = 0.04)]. Trial 2 was also significantly higher than Trial 4 (*p* = 0.003). A planned *post hoc* comparison of Trial 1 confirmed no difference in the initial investigation of the new stimulus mouse type (WT vs. Avpr1b^Grin1+/−^, *p* = 0.39, WT vs. Avpr1b^Grin1−/−^ *p* = 0.52). Together these indicate a short-term behavioral habituation to the stimulus animal. A robust dishabituation response was observed upon the presentation of the novel stimulus mouse revealed by comparing Trial 4 and the Novel Trial (*p* < 0.001).

### Non-social Memory Testing

#### Object Habituation-Dishabituation

All groups performed similarly in the object habituation-dishabituation test (Figure 9B). There was no difference in the initial interaction time. Two-way repeated measures ANOVA (Trial × Genotype) revealed a significant effect of Trial (*F*_(2.2,53.1)_ = 7.3, *p* < 0.001), but no effect of genotype (*F*_(2,24)_ = 0.8, *p* = 0.4), and no interaction (*F*_(8,96)_ = 1.4 *p* = 0.2). *Post hoc* Tukey’s multiple comparisons revealed Trial 1 sniffing duration was significantly higher than later trials [compared with Trial 3 (*p* = 0.001), Trial 4 (*p* < 0.001)]. Together these indicate a short-term behavioral habituation to the presented object. A dishabituation response was observed upon the presentation of the novel object revealed by comparing Trial 4 and the Novel Trial, although this response was not significant (*p* = 0.08).

#### Olfactory Habituation-Dishabituation

All groups performed similarly in the olfactory habituation-dishabituation test (Figure 9C). There were no differences in the initial interaction times. Two-way repeated measures ANOVA (Trial × Genotype) revealed a significant effect of Trial (*F*_(8,192)_ = 6.1, *p* < 0.001), but not genotype (*F*_(2,24)_ = 0.4, *p* = 0.7). *Post hoc* Tukey’s multiple comparisons revealed significant decreases between the initial almond presentation and subsequent trials (Almond Trial 2, *p* = 0.003, Almond Trial 3, *p* = 0.003). Significant dishabituation was observed between the third almond presentation and the first urine presentation (*p* = 0.04).

### Anxiety-Like Behaviors

#### Perseverative and Escape Behaviors

**Figure 10:**
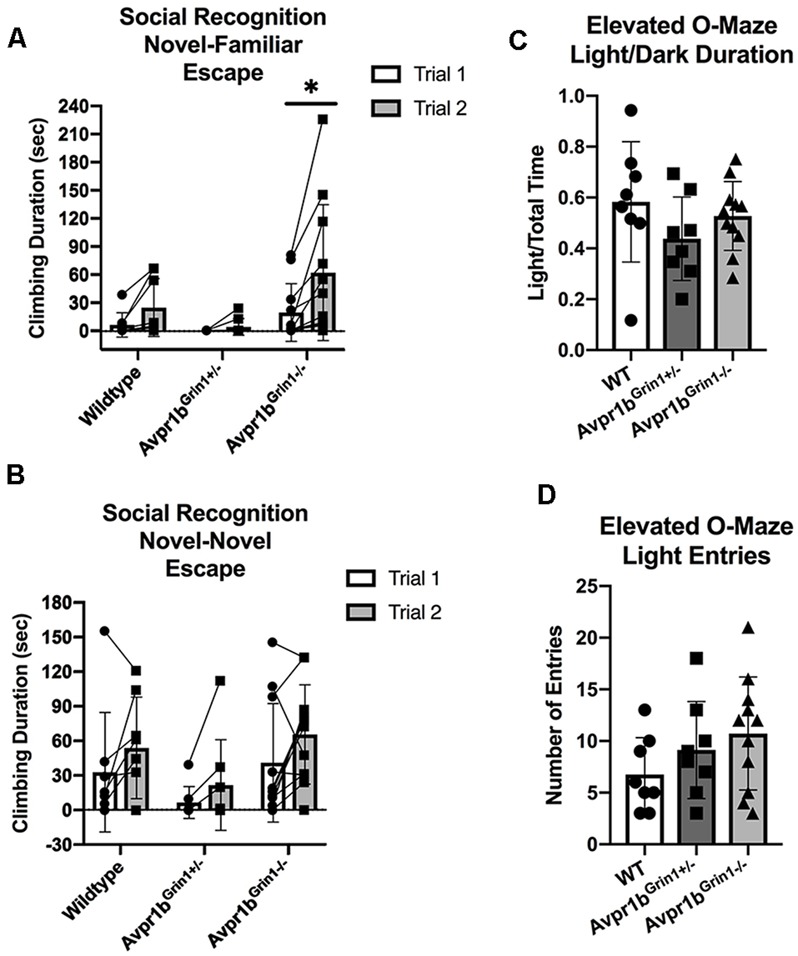
Anxiety-like behaviors are not disrupted in Avpr1b^Grin1−/−^ mice. **(A,B)** Escape behavior (climbing behavior) was measured during the social recognition behavioral tests. **(A)** Two-way repeated measures ANOVA revealed that during the social recognition novel-familiar test there was a significant effect of Trial (*F*_(1,24)_ = 10.23, *p* = 0.004), but no significance for genotype (*F*_(1,24)_ = 3.1, *p* = 0.059) or interaction (*F*_(2,24)_ = 3.0, *p* = 0.06). Avpr1b^Grin1−/−^ mice had a significant increase in climbing (Sidak’s multiple comparison adjusted *p* = 0.001). During the social recognition novel-novel test, there was a significant effect of Trial (*F*_(1,24)_ = 8.0, *p* = 0.0002), but no longer any effect of genotype (*F*_(2,24)_ = 2.4, *p* = 0.11). **(C)** Elevated O-Maze. Ratio of duration spent on the light arms. One-way ANOVA indicated no group differences. **(D)** Locomotor activity indicated by number of entries into either light arm. One-way ANOVA indicated no group differences. All data are presented as individual responses, with means ± SD overlaid.

Since NMDA function in the forebrain has been tied to symptoms observed in animal models of schizophrenia (Uno and Coyle, 2019), we analyzed perseverative and escape/avoidance behaviors during the social recognition tests. Perseverative behaviors, including digging in the bedding and rearing in the cage, showed no significant differences between the groups. Specifically during the social recognition novel-familiar test, two-way repeated ANOVA reported no difference in genotype for digging (*F*_(2,24)_ = 0.5, *p* = 0.58) or rearing (*F*_(2,24)_ = 0.3, *p* = 0.72). To assess escape/social avoidance behaviors, climbing behavior was defined as climbing up the edge of the behavioral chamber with no access to the presented stimuli (Figures 10A,B). Two-way repeated measures ANOVA revealed that during the social recognition novel-familiar test there was a significant effect of Trial (*F*_(1,24)_ = 10.23, *p* = 0.004), and but not for an interaction (*F*_(2,24)_ = 3.0, *p* = 0.06) or for genotype (*F*_(1,24)_ = 3.1, *p* = 0.059). Avpr1b^Grin1−/−^ mice had a significant increase in climbing (Sidak’s multiple comparison adjust *p* = 0.001), while WT (*p* = 0.3) and Avpr1b^Grin1+/−^ (*p* = 0.9) did not differ between trials. During the social recognition novel-novel test, there was a significant effect of Trial (*F*_(1,24)_ = 8.0, *p* = 0.0002), but no longer any effect of genotype (*F*_(2,24)_ = 2.4, *p* = 0.11) on climbing behavior.

#### Elevated O-Maze

We measured anxiety-like behavior using the elevated O-maze (Figures 10C,D). There were no significant differences in the number of entries (*F*_(2,24)_ = 1.6, *p* = 0.21), duration spent in the open arms (*F*_(2,24)_ = 1.3, *p* = 0.27), or duration of stretching into the open arm (Welch’s *W* = 2.8, *p* = 0.10). Locomotor activity was unaffected as measured by distance traveled during the test (*F*_(2,24)_ = 0.66, *p* = 0.53).

## Discussion

We describe a new transgenic mouse line that placed Cre recombinase expression under the control of the Avpr1b gene promoter. By using this approach, we sought to avoid the physiological and behavioral effects that can accompany unknown genomic insertion sites and off-target Cre-expression that are common in many BAC transgenic lines (Heffner et al., 2012; Harno et al., 2013). Gross physiological development was normal in this strain with litter number, sex ratio, and adult weight comparable to C57Bl/6J mice in our lab. We compared heterozygous Avpr1b-Cre mice to their WT siblings and found no differences in social approach, interaction or recognition behaviors, confirming that the Cre insertion did not cause off-target behavioral effects relevant to this study in this strain. Avpr1b and Cre mRNA localized to CA2 neurons, while there was minimal expression within the dentate gyrus, CA1 or CA3. Using a transgenic constitutive labeling approach (i.e., cross with a Cre-activatable fluorescent marker mouse line), we observed labeled neurons within the dorsal and ventral CA2, olfactory bulbs, caudate-putamen. Previous work has demonstrated Avpr1b gene expression in the paraventricular nucleus of the hypothalamus and the medial nucleus of the amygdala; however, although it is occasionally observed, we do not observe consistent expression in either the PVN or MEA. We, therefore, speculate there may be some physiological state that induces expression, but this is currently under investigation. Although we propose this model will be useful for CA2 neuronal targeting, caution should be taken with constitutive approaches. For example, we do not see Avpr1b expression by *in situ* hybridization histochemistry in the adult caudate-putamen, presumably because transient expression of Avpr1b driving Cre earlier in development activated the fluorescent marker gene.

Crossing this Avpr1b-Cre line with a transgenic line with a floxed Grin1 gene resulted in the removal of functional NMDA receptors from Avpr1b neurons. We assessed this line in a battery of behavioral tests measuring memory, social and emotional behaviors. Surprisingly, a lack of NMDA in Avpr1b (including most CA2) neurons showed a minimal effect on any of the behaviors assessed in this study. This indicates that NMDA-dependent mechanisms may not be required for Avpr1b-dependent behaviors.

Avpr1b KOs show a robust deficit in aggressive social behavior in both males and females (Wersinger et al., 2002, 2004). Replacement of Avpr1b into the dorsal CA2 of Avpr1b KO males results in the rescue of aggressive behavior (Pagani et al., 2015). Furthermore, the acute activity of dorsal CA2 neurons has also recently been shown to be required for typical expression of aggression (Williams Avram et al., 2016; Leroy et al., 2018). The exact mechanism through which Avpr1b activity may be contributing to the role of CA2 in aggression remains poorly understood. Aggression is often considered an innate behavior but has an important learned component. Aggressive behavior skills are developed by male mice during adolescence and change based on experience. NMDA receptor activity within the ventral hippocampus, where there are abundant Avpr1b neurons, is critical for typical aggressive behavior expression (Chang et al., 2018). Surprisingly, we do not observe any differences in aggressive behavior in the Avpr1b^Grin1−/−^ mice, either in the likelihood to exhibit the behavior or in the duration or frequency of attacks.

Avpr1b KO mice have deficits in short-term social recognition and social habituation (Wersinger et al., 2002; Stevenson and Caldwell, 2012; Williams Avram and Cymerblit-Sabba, 2017). Similarly, disruption of CA2 neuronal activity chronically and acutely can inhibit short-term social recognition (Hitti and Siegelbaum, 2014; Stevenson and Caldwell, 2014; Meira et al., 2018). Although the activity of pyramidal neurons of the CA2 is required for social recognition, NMDA-dependent molecular plasticity within the pyramidal neurons may not be required. Similar to our findings, Finlay et al. (2015) show that genetic removal of NMDA receptors from the dorso-lateral hippocampus (inclusive of CA2 and CA3 neurons) in adulthood resulted in no effect on social novelty preference, which requires social memory.

The apparent lack of the requirement for NMDA receptors in postnatal development or acutely in adulthood in CA2 neurons for social recognition is in contrast to recent data demonstrating this requirement in CA1 and CA3 neurons. Constitutive removal of NMDA receptors from CA3 neurons [using the glutamate receptor, ionotropic, kainate4 (Grik4)-Cre line crossed to the same floxxed Grin1 line] results in deficits in short-term social recognition and social novelty preference while leaving object recognition intact (Chiang et al., 2018). The same study showed that constitutive removal of NMDA receptors from CA1 neurons [using the calcium/calmodulin-dependent protein kinase II alpha (*Camk2a*)-Cre line crossed to the same floxxed Grin1 line] showed no deficit in social recognition. However, if the removal of NMDA receptors in the CA1 is induced during adulthood, mice do exhibit deficits in the short-term and long-term social and object recognition (Jacobs and Tsien, 2017). Taken together, these data suggest that NMDA receptor activity in the hippocampus may play a larger role in social recognition storage or retrieval.

With the exception of a subtle effect in temporal order memory (DeVito et al., 2009), Avpr1b KO mice do not show deficits in non-social memories (Wersinger et al., 2002, 2004). Similarly, we did not observe any object or olfactory memory deficits in Avpr1b^Grin1−/−^ mice. As noted above, NMDA signaling in other regions of the hippocampus have been shown to be critical for non-social memories. The constitutive CA1 Grin1 KOs show a strong deficit in object memory and olfactory memory. Interestingly, these deficits are rescued by exposure to enriched conditions (Rampon et al., 2000). Similarly, CA3 Grin1 KOs exhibit deficits in acquisition and recall of associative memories (Nakazawa et al., 2002). Notably, inducible removal of Grin1 from CA1 neurons during adulthood results in deficits in the acquisition of spatial memory (Shimizu et al., 2000), but no effect on recall. This deficit is only observed in inducible models as constitutive removal of Grin1 resulted in no alteration in memory performance (Mei et al., 2011). For CA2 neurons, both constitutive (our data) and virally-mediated removal during adulthood, result in typical performance of social memory tasks. NMDA receptors may have a more important role in other tasks these neurons are known to be involved with, such as subtle novelty detection, detection/processing of time, and fear learning (Mankin et al., 2015; Alexander et al., 2016, 2019). Alternatively, the role of NMDA receptors in adult CA2 neurons may be dependent on critical developmental environmental exposures. These data suggest a complex context- and memory-type specific role for NMDA receptors within hippocampal pyramidal neurons that will require more attention.

Our data suggest that Avpr1b (and thus most CA2) neurons do not require NMDA receptors to perform their role in social recognition. It is possible that the constitutive removal of NMDA receptors results in compensatory changes in these, or other, neurons to allow for performance of these important behavioral functions. For example, it is known that the Avpr1b-mediated enhanced EPSC response observed in dorsal CA2 neurons can be blocked by NMDA receptors blockade, but also blocked by interrupting other intracellular calcium signaling mechanisms. Avpr1b-expressing neurons may have adapted functions of other calcium signaling mechanisms, such as calcium permeable AMPA receptors, or voltage-gated calcium channels to permit the typical response. CA2 neurons have distinct calcium buffering properties that are regulated by the regulator of G protein signaling 14 (RGS14) protein (Evans et al., 2018) and are uniquely encased with a perineuronal net that regulates plasticity of CA2 synapses (Carstens et al., 2016). It remains unclear how any of these molecular signals may rely on developmental or current expression of NMDA receptors to regulate their expression.

Furthermore, although pyramidal CA2 neurons do not respond to standard long-term potentiation inducing protocols, it was recently observed that input-timing dependent plasticity can occur, but that this change in pyramidal neuron response is driven by long-term depression of adjacent parvalbumin (PV) interneurons in the CA2 region (Dudek et al., 2016; Leroy et al., 2017). Blocking delta opioid receptors, which are found on PV interneurons in the CA2, was able to block the depression in the PV interneurons, the potentiation in the pyramidal neurons and short-term social memory (Leroy et al., 2017). These elegant studies provide a potential mechanism through which CA2 neurons may not require NMDA-dependent plasticity.

Alternatively, Avpr1b neurons may rely on a form of presynaptic plasticity. There are several forms of presynaptic plasticity that do not rely on NMDA receptors (Yang and Calakos, 2013). Vasopressin, acting through Avpr1b, was recently shown to underlie presynaptic plasticity in dCA2 neuronal projections (Leroy et al., 2018). Interestingly, this presynaptic plasticity appears to be projection specific as it was observed in the dorsal lateral septum, where it played as critical role in aggressive behavior, but not in the dorsal CA1 projections. As social memory engrams are formed in the ventral CA1, and chemogenetic inactivation of dCA2 neuronal fibers in vCA1 results in memory deficit (Okuyama et al., 2016; Meira et al., 2018), it would be interesting to test the presynaptic plasticity mechanism of Avpr1b neuronal projections to the ventral CA1 region.

Reductions in NMDA signaling either pharmacologically or genetically have generally been found to disrupt social interactions. NMDA antagonists such as phencyclidine (PCP) can reduce social interactions and social recognition performance (Neill et al., 2010; Zimnisky et al., 2013). Furthermore, mice with genetically reduced NMDA receptor levels have reduced social interaction (Mohn et al., 1999; Duncan et al., 2004, 2009; Halene et al., 2009; Mielnik et al., 2014). Genetic removal of NMDA receptors throughout the forebrain during adulthood results in impairments in social approach, as well as reduced long-term social recognition (Jacobs and Tsien, 2017). Similarly, removal of Grin1 from dorso-lateral hippocampus in adulthood results in decreases in social approach (Finlay et al., 2015). However, we observed no effect on social interaction in any of our tests. Our data are in agreement with previous studies of Avpr1b KO mice, which do not show reductions in social interaction or social approach behavior (Wersinger et al., 2002; Yang et al., 2007). Furthermore, no studies investigating the chronic or acute disruption of CA2 neurons have reported any changes in social interactions or social approach (Hitti and Siegelbaum, 2014; Stevenson and Caldwell, 2014; Piskorowski et al., 2016; Smith et al., 2016; Leroy et al., 2017; Meira et al., 2018).

## Conclusion

To our knowledge, this is the first report to indicate that NMDA function is not required in neurons known to play a critical role in memory performance. Given the lack of behavioral effects, it is tempting to conclude that NMDA receptors on Avpr1b neurons in the hippocampus have no role in social aggression, approach or social memory. However, social approach, social recognition, and aggression performance can be disrupted by manipulation of several distributed brain areas, ranging from olfactory bulb to hypothalamus to prefrontal cortex, suggesting many cognitive processes are involved in the calculations to perform aggressively or store a social memory. Furthermore, the salience of the social cues may play an important role in the modulation of decision-making and memory acquisition circuits, indicating the possibility that NMDA receptor function in Avpr1b neurons may be acting in a more modulatory role. Further investigation using our novel Avpr1b-Cre transgenic mouse line will help elucidate mechanisms within Avpr1b neurons and how they contribute to social aggression and memory.

## Data Availability Statement

The datasets generated for this study are available on request to the corresponding author.

## Ethics Statement

The animal study was reviewed and approved by the Animal Care and Use Committee of the National Institute of Mental Health.

## Author Contributions

SW: experimental design, behavioral data analysis, image data collection and analysis, manuscript preparation. H-JL: creation of transgenic mouse. JF: behavioral data collection and analysis. AC-S: experimental design, data analysis, and manuscript preparation. AS: experimental design and data analysis. MV: behavioral data collection and analysis. JS: molecular data collection. MG: behavioral data collection and analysis. S-HL: experimental design and cellular data collection. NC: cellular data collection. MS: behavioral data collection and analysis. RC: behavioral data collection and analysis. WY: experimental design, data analysis, and manuscript preparation.

## Conflict of Interest

The authors declare that the research was conducted in the absence of any commercial or financial relationships that could be construed as a potential conflict of interest.

## Footnotes

^1^http://www.jwatcher.ucla.edu/
